# Reusable and Interface-Confined Photothermal Electrospun Nonwovens for the Selective Removal of Polymeric Coatings

**DOI:** 10.1021/acsami.6c04133

**Published:** 2026-06-24

**Authors:** Francesca Ramacciotti, Arianna Menichetti, Maddalena Redi, Emilio Catelli, Giulia Di Cara, Giorgia Sciutto, Maria Letizia Focarete, Ilaria Degano, Laura Cartechini, Francesca Rosi, Sara Mattana, Martina Alunni Cardinali, Marco Montalti, Chiara Gualandi, Silvia Prati

**Affiliations:** † Department of Chemistry “Giacomo Ciamician”, 9296 University of Bologna, Bologna 40129, Italy; ‡ Department of Chemistry “Giacomo Ciamician”, University of Bologna, Rimini 47922, Italy; § Department of Chemistry “Giacomo Ciamician” and INSTM UdR of Bologna, University of Bologna, Bologna 40129, Italy; ∥ Department of Chemistry and Industrial Chemistry, University of Pisa, Pisa 56124, Italy; ⊥ National Research Council, Institute of Chemical Science and Technologies “G. Natta” (CNR-SCITEC), Perugia 01623, Italy; # Department of Chemistry, Biology, and Biotechnology, 9309 University of Perugia, Perugia 01623, Italy; ∇ Interdepartmental Center for Industrial Research on Advanced Applications in Mechanical Engineering and Materials Technology, CIRI-MAM, University of Bologna, Bologna 40123, Italy

**Keywords:** electrospinning, photothermal effect, coating removal, street art, hyperspectral imaging

## Abstract

The selective removal of polymeric coatings from multilayer films can be challenging when the outer layer is highly insoluble and chemically similar to the underlying layers. In this context, photothermal materials represent a promising strategy for locally activating polymer softening. In this paper, we studied heat delivery at solid–solid interfaces in a bioderived electrospun nonwoven incorporating melanin nanoparticles as a photothermal agent for the selective removal of polymeric coatings applied to a multilayer system. Through controlled solvent loading and light-induced photothermal heating, a localized temperature modulation at the material–substrate interface can be achieved. A dedicated experimental setup integrating thermal imaging and thermocouple measurements enables direct quantification of interface temperatures under operational conditions. As a proof of concept, the method has been applied to remove a highly insoluble cross-linked alkidic layer applied on an underlying acrylic or alkyd polymer coatings. The efficacy and selectivity of the proposed method have been assessed using a multianalytical protocol that combines imaging techniques with chemometric analysis, HPLC-DAD, and Brillouin microspectroscopy. The synergistic action of photothermally generated heat and the solvent allowed reducing both the amount of solvent used and the application time, while preserving the chemical composition and viscoelastic properties of the underlying layers. Additionally, the electrospun nonwovens remained effective after three cleaning cycles. These results demonstrate that photothermal electrospun nonwovens provide a versatile platform for interface-confined thermal activation, offering new opportunities for selectively removing organic coatings from multilayer systems.

## Graphical abstract

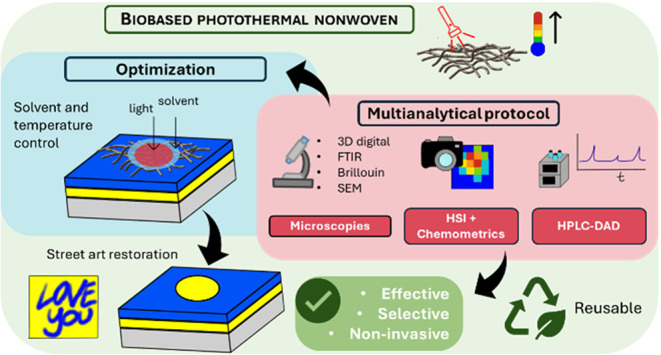

## 1 Introduction

The selective removal of polymeric coatings from multilayer films is a critical issue across several industrial and technological sectors that require coating removal in regular maintenance cycles, either locally for repairs or completely for refurbishment. Some examples can be found in the aerospace industry, where the controlled removal of multilayer systems (primer, adhesives, topcoat) from components made of alloys or composite materials is frequent.^1^ In electronics and microelectronics photoresists or protective polymer layers must be efficiently removed without damaging the metallized or dielectric layers below. The removal of antifouling or protective coatings without damaging anticorrosion primers is important in the marine industry in the conservation.^2^ In restoration of cultural heritage the removal of aged varnishes or graffiti with high selectivity to avoid altering original layers is imperative.^3^ Indeed, the selective removal of polymeric coating is particularly challenging when a specific layer must be removed without damaging the underlying ones, when the unwanted layer is highly insoluble and/or when the coating to be removed is chemically similar to the underlying polymeric layers, as in multilayer organic systems where differences in solubility and adhesion are insufficient to ensure selective removal, often leading to swelling, dissolution, or leaching of the substrate.^4^ Also, the cleaning technology should avoid substances that cause significant environmental impacts and waste.

We recently proposed a sustainable electrospun nonwoven mat made of pullulan, a water-soluble natural polysaccharide, combined with melanin nanoparticles, demonstrating the outstanding photothermal properties of the resulting material and its high potential for removing cross-linked alkyd spray paints from stone substrates.^5^ Melanin is a dark brown natural pigment that, upon light irradiation, preferentially undergoes nonradiative relaxation, converting absorbed energy into heat.^6,7^ Electrospinning is a versatile technique widely studied in various fields, from tissue engineering^8^ to sensors^9^ and to air^10^ and wastewater filtration.^11^ Ultrathin fibers are produced via an electrodynamic process, yielding nonwoven nanostructured materials with controlled properties, including fibers, membranes, and bundles.^12,13^ The high porosity and large specific surface area of electrospun nonwovens confer excellent sorbent and retentive capabilities, making them particularly suitable as confined carriers for active agents.^5,6^ The developed material^5^ combines the retentive properties of electrospun fibers^6^ and the photothermal features of melanin;^14^ thus, the synergic action of the confined solvent and of the photothermally generated heat allows to soften locally the alkydic insoluble polymeric layer, enabling efficient removal within short application times and with limited solvent consumption.

Building on our previous work,^5^ the present study aims to further characterize photothermal electrospun materials based on melanin and pullulan, with particular emphasis on elucidating their photothermal behavior and their ability to enable selective cleaning at polymer–polymer interfaces. Specifically, we investigate the removal of cross-linked alkyd overpaintings from underlying acrylic and alkyd layers, a representative and highly challenging multilayer polymeric system. Such a situation may be encountered in the context of urban art and more generally, urban furniture and polymer-based decorative surfaces, where the selective removal of vandalic overpaintings represents one of the most complex scenarios, as both the original artwork and the unwanted layers often rely on chemically similar polymeric binders.^3,15–17^ In this context, selectivity toward the target layer and preservation of the underlying matrix are of paramount importance. In particular, alkyd paints used in spray coatings are highly insoluble due to their cross-linked structure, making their selective removal extremely difficult using conventional physical or chemical methods.^17,18^ Abrasive techniques or solvent application by swabs are often ineffective or excessively invasive. At the same time, alternative approaches, such as laser cleaning, require careful case-by-case optimization and may still pose risks to the layers to be preserved.^19–25^ As a result, significant research efforts have been devoted to the development of advanced materials and methodologies capable of delivering efficient yet minimally invasive cleaning action, including solvent-retaining gels,^26–33^ and, more recently, nonwoven polymeric systems used either in combination with gels and microemulsions or as independent retentive platforms.^5,34–36^

The present work focuses on a detailed investigation of the photothermal behavior of the nonwoven, evaluating its photothermal efficiency and developing an experimental setup integrating thermal imaging and thermocouple measurements to directly monitor the temperature reached at the interface between the electrospun nonwoven and the polymeric coating during treatment. In parallel, the optimal solvent loading was evaluated as a function of the temperature required to promote swelling of the cross-linked alkyd layer. Cleaning performance and selectivity were assessed through a comprehensive multianalytical protocol that combined imaging techniques and chemometric analysis with high-performance liquid chromatography and Brillouin light scattering microspectroscopy. The results demonstrate not only the effectiveness of the proposed approach, but also its minimal chemical and mechanical impact on the underlying layers. Furthermore, the reusability of the electrospun nonwoven mats across multiple cleaning cycles highlights the potential of this material platform for sustainable, controlled surface-coating removal.

## 2 Experimental Section

### 2.1 Materials

γ-valerolactone (GVL, Reagent plus, ≥99%) was purchased from Sigma-Aldrich, and pullulan was purchased from TCI Europe. Melanin was obtained from cuttlefish ink by centrifugation.^5,37^ The alkyd sprays “94, RV 154 Azul Tornado/Tornado Blue” (AlkB) and “94, RV 1021 Amarillo Claro/Light Yellow” (AlkY) paints were purchased from Montana Colors, and the acrylic spray “Flame Orange, Zinc Yellow” (AcrY) was purchased from Molotow. The yellow pigment PY74 standard was purchased from Kremer Pigmente, and dimethyl sulfoxide (DMSO; ≥99.9%, HPLC grade) was purchased from J.T. Baker.

### 2.2 Fabrication of Nonwovens

A laboratory electrospinning unit (Spin-bow Lab Unit, Spinbow S.r.l., Italy) was used to produce the nonwoven mats. The setup consists of a syringe filled with the polymer solution, connected to a blunt-tipped steel needle (internal diameter: 0.51 mm) via a polytetrafluoroethylene (PTFE) tube. An aluminum plate, covered with a PTFE mask (either 10 × 10 cm^2^ or 5 × 5 cm^2^), served as the collector. The needle-to-collector distance was fixed at 18 cm, the applied voltage ranged between 24 and 25 kV, and the polymer solution flow rate was maintained at 0.8 mL h^–1^. Two types of electrospun nonwovens were fabricated: the PULL sample was prepared from a 19% w/v pullulan solution in water, while the PULL_M sample was obtained by electrospinning a 19% w/v pullulan solution containing melanin nanoparticles dispersed at 18 mg mL^–1^ in water. Before electrospinning, the melanin dispersion was sonicated for 10 min to prevent nanoparticle aggregation.

### 2.3 Mock-Up Preparation

The concrete supports were prepared by mixing 2.5 parts mix sand, consisting of 2 parts natural fine sand (Sabbia AXTON fine naturale di fiume) and 1 part mixed coarse sand (Sabbia AXTON vagliata); 1 part cement (i.work TECNOCEM A-LL 32,5 R); and 0.65 to 1 parts water. The samples were polished with 180-grit sandpaper. The layer simulating the street artwork was created by spraying three consecutive AlkY or AcrY spray-can layers. The spray cans were well shaken and kept at 15–20 cm away from the substrate. The skinny-type caps were employed (0.2–2 mm). The mock-ups were aged in a Solar Box (Q-Sun Xe-1 Xenon Test Chamber (Q-Lab Corporation, UK)) by exposing the samples at an irradiance of 0.68 W m^–2^ under Daylight Q filters at 50 °C for 1500 h. These aging conditions were selected to simulate direct sunlight exposure and induce a mild degradation.^20^ The AlkB layer mimicking the vandalic act was then applied using the same procedure described above for the yellow varnishes. Then, the mock-ups were placed in an oven at 70 °C for 48 h to ensure complete drying and cross-linking.

### 2.4 Electrospun Mat Photothermal Properties Characterization

The fabrics were characterized by Scanning Electron Microscopy (SEM) and Transmission Electron Microscopy (TEM) in a previous study where a characterization of mat photothermal properties was performed using various set-ups.^5^ In this work, further photothermal analyses were performed by irradiating the mat with an LED at 660 nm (LZ1–10R2, Osram) (Figure S2). The LED, equipped with a lens (Thorlabs), irradiated an area of 5 × 5 mm^2^ and was placed 6 cm away from the sample. The LED irradiance can be adjusted by tuning the applied current, corresponding to the following irradiances: 223 W m^–2^ (0.1 A), 435 W m^–2^ (0.2 A), 648 W m^–2^ (0.3 A) and 858 W m^–2^ (0.4 A). The effect of the solvent on temperature was evaluated by irradiating a 1 × 1 cm^2^ mat loaded with different amounts of GVL at the following S/M ratios: 1 μL mg^–1^, 2 μL mg^–1^, 3 μL mg^–1^, and 4 μL mg^–1^. The temperature of the irradiated area at 648 W m^–2^ was monitored with a thermal camera (Optris Xi 400, IR camera, 80HZ frame rate, and 382 × 288 pixels of optical resolution) using the Optris PIX connect software. Moreover, the T at the interface between the irradiated fabric and the mock-up surface was measured with a thermocouple (RS pro, RS-14 digital multimeter, with a K-type thermocouple), for irradiances 223 W m^–2^, 435 W m^–2^, 648 W m^–2^, and 858 W m^–2^. Photothermal efficiency evaluation was performed on four melanin nonwovens (PULL_M) wet by GVL 2 μL mg^–1^, using a PULL nonwoven wet by GVL 2 μL mg^–1^ as a reference. The absorbance of the nonwovens was evaluated by measuring transmittance and reflectance using a PerkinElmer Lambda45 spectrophotometer equipped with the integrating reflectance sphere RSA-PE-20.

### 2.5 Evaluation of the Removal Performance

Each application test was performed with an electrospun nonwoven 1 × 1 cm^2^. Each mat was weighed three times and loaded with GVL solvent either at 2 μL mg^–1^ or 4 μL mg^–1^. During all the removal tests, samples were irradiated either at 223 W m^–2^ or at 648 W m^–2^, named respectively “low” and “high” irradiances, for 2 min. After each application, three dry cotton swabs were gently rolled over the area to remove the blue alkyd varnish residues.

#### 2.5.1 Evaluation of Removal Efficacy

3D images of the samples in reflected light were acquired using the digital microscope Hirox RX-100 at magnifications of 40×, 320×, and 500×. The instrument is equipped with a 2.38-megapixel CMOS Sensor and can work with two objectives, offering magnification ranging from 6× to 2500× for 2D and 3D acquisitions. The head is mounted on a vertical motorized stage reaching a maximum *Z*-resolution of 0.05 μm and an *X* and *Y* maximum resolution of 0.04 μm at the highest magnification.

The areas after removal were analyzed with a spectral imaging system (Camera Specim IQ) operating in the visible–near-infrared range (400–1000 nm), purchased by Specim Finland, with a of distance of 16 cm, resulting in a pixel size of approximately 300 μm × 300 μm. After the acquisition, the 3D data array was submitted to preprocessing transformations in order to remove unwanted instrumental spectral variations such as baseline shifts and drifts and not relevant information. In particular, the data array was spectrally cropped to the spectral range of interest (450–900 nm), and a first derivative was computed using the Savitzky-Golay algorithm (polynomial order: 3, data point window size: 11). Image segmentation was then performed to classify pixels into clusters related to different degrees of removal for each treated area. To this aim, principal component analysis, known for its efficiency in data reduction, was implemented on the data and the first score map (PC1) was considered for data segmentation by clustering it into three spatial clusters on the basis of the frequency distribution of PC1 score values. A false-color RGB image was then created to provide a quick visualization of the spatial segmentation process. In addition, average reflectance spectra were extracted from each segmented cluster to evaluate their spectral differences.^38^ Then, the pixels in each channel were counted, providing a percentage value normalized by the dimension of the treated area.

#### 2.5.2 Evaluation of the Selectivity and Invasiveness toward the Layers to be Preserved

The method selectivity toward the removed layer was investigated by analyzing the possible paint residues collected with the swabs after removal, and in particular, the presence of yellow pigment PY74 was investigated via HPLC-DAD on DMSO extracts. For the quantification of PY74, a calibration curve was obtained by analyzing five standard solutions at 10, 5, 1, 0.5, and 0.1 ppm, prepared by dissolving a PY74 standard (Kremer Pigmente) in dimethyl sulfoxide (DMSO; HPLC grade, J.T. Baker) (See Supporting Information for further details). All samples underwent multiple solvent extractions in DMSO in an ultrasonic bath at 60 °C for 20 min. Before HPLC-DAD injection, the extracts were filtered through PTFE filters (4 mm thickness and 0.45 μm pore diameter). The HPLC-DAD system used consists of a PU-2089 quaternary pump with a degasser, an AS-950 autosampler, and an MD-2010 spectrophotometric diode-array detector (DAD) operating in the 200–650 nm wavelength range with 4 nm resolution and a 0.2 s acquisition rate. All modules were from Jasco International Co., Tokyo. JASCO ChromNav software was used for HPLC-DAD data acquisition and analysis. Chromatographic separation was performed using a Poroshell 120 EC-C18 analytical reversed-phase column (3.0 mm × 75 mm, particle size 2.7 μm), equipped with a Poroshell 120 EC-C18 guard column (3.0 mm × 5 mm, particle size 2.7 μm). Both the column and the guard column were from Agilent Technologies (Palo Alto). The column was maintained at 30 °C during the separation. The mobile phase eluents used were: A = water with 1.0% v/v of formic acid (FA); B = acetonitrile with 0.3% v/v of formic acid (FA). The separation was performed using the following chromatographic gradient: 5% B for 2.6 min, then to 50% B in 13.0 min, to 70% B in 5.2 min, to 100% B in 6.2 min, and then hold for 3.0 min; re-equilibration took 11 min. All solvents and mobile phase modifiers (*i.e.*, formic acid) were purchased from Sigma-Aldrich (USA) at HPLC grade. The experiments were performed at a flow rate of 0.6 mL min^–1^. Injection volumes were 10 μL for all the extracts.

μATR-FTIR spectroscopy was performed with a Thermo Nicolet (Thermo Fisher Scientific, Waltham, MA, USA), iN10MX imaging microscope fitted with a mercury–cadmium-telluride detector cooled with liquid nitrogen. Measurements were performed using a slide-on ATR objective equipped with a conical germanium crystal in the range 4000–675 cm^–1^, at a spectral resolution of 4 cm^–1^ with 64 scans and an optical aperture of 150 μm × 150 μm. The analysis was performed before removal to collect reference spectra of the materials and after removal to evaluate the presence of varnish residues. Three spectra were collected in each area.

The Brillouin light scattering microspectroscopic setup consists of a 532 nm single-mode solid-state laser (Oxxius, Optoprim), which is focused onto the sample surface by a microscope objective (20×, NA = 0.42, Mitutoyo). The backscattered light is directed to a high-contrast multipass tandem Fabry–Perot interferometer (TFP-2 HC, JRS Scientific Instruments). The sample is mounted on an *XYZ* translation stage for single-point and mapping experiments.^39^ Each Brillouin spectrum was collected using 1300 scans, with input and output pinhole apertures of 150 and 450, respectively, and a mirror distance of 5 mm. The laser power at the sample was set to <1 mW to avoid local photodamage. The frequency shift of the Brillouin peaks was determined using the first spectral moment, calculated by averaging the Stokes and anti-Stokes contributions within the frequency ranges of 10–18 GHz for acrylic varnish and 11–20 GHz for alkyd varnish, as previously described.^40^ Indeed, the utilization of spectral moments, which do not depend on the arbitrary selection of a fitting function, has been demonstrated to reduce the error in the estimation of the average Brillouin frequency shift in the case of peak broadening from multiple scattering phenomena, as observed in the present samples.

### 2.6 Reusability of the Nonwovens

During the reuse cycles, the LED irradiance was set at 223 W m^–2^; in each cycle, each mat was loaded with 2 μL mg^–1^ of solvent and was irradiated for 1 min. For each cycle, the temperature recorded during irradiation was averaged. Then, temperatures obtained over 3, 5, and 10 cycles were averaged. The mats after the reuse cycles were characterized using Scanning electron microscope (SEM) and μFTIR. SEM (Leica Cambridge Stereoscan 360) was performed on the mats after 3, 5, and 10 removal applications at 20 kV accelerating on gold-sputtered samples. The distribution of the nanofiber diameters was measured on at least 200 fibers and was obtained as average diameter ± standard deviation. μFTIR mapping in reflection mode spectroscopy was performed with a Thermo Nicolet (Thermo Fisher Scientific, Waltham, MA, USA), iN10MX imaging microscope fitted with a mercury–cadmium-telluride detector cooled with liquid nitrogen. Measurements were performed in the range 4000–675 cm^–1^, at a spectral resolution of 4 cm^–1^ with 16 scans, an optical aperture of 300 μm × 300 μm, a step of 300 μm and the background acquisition was performed every 15 min.

## 3 Results and Discussion

### 3.1 Electrospun Mat Photothermal Properties Characterization

γ-valerolactone (GVL), a green solvent that has been demonstrated to be effective in swelling cross-linked alkyd spray paints, was used in this work.^5^ Since high amounts of solvent may dissipate the photothermally generated heat, resulting in an adverse effect on the removal efficacy, first tests have been performed to study the solvent effect on the temperature reached by the nonwoven upon irradiation in order to identify the most suitable solvent-to-mat ratio. To this purpose, the mats have been impregnated with different amounts of GVL, expressed as solvent-to-mat ratio (*S*/*M* in μL mg^–1^), irradiated at constant irradiance corresponding to the conditions employed in ref 5 (648 W m^–2^) and with a round spot size of 7 mm of diameter, while monitoring the temperature on the irradiated side of the mats (Figure 1).

**Figure 1:**
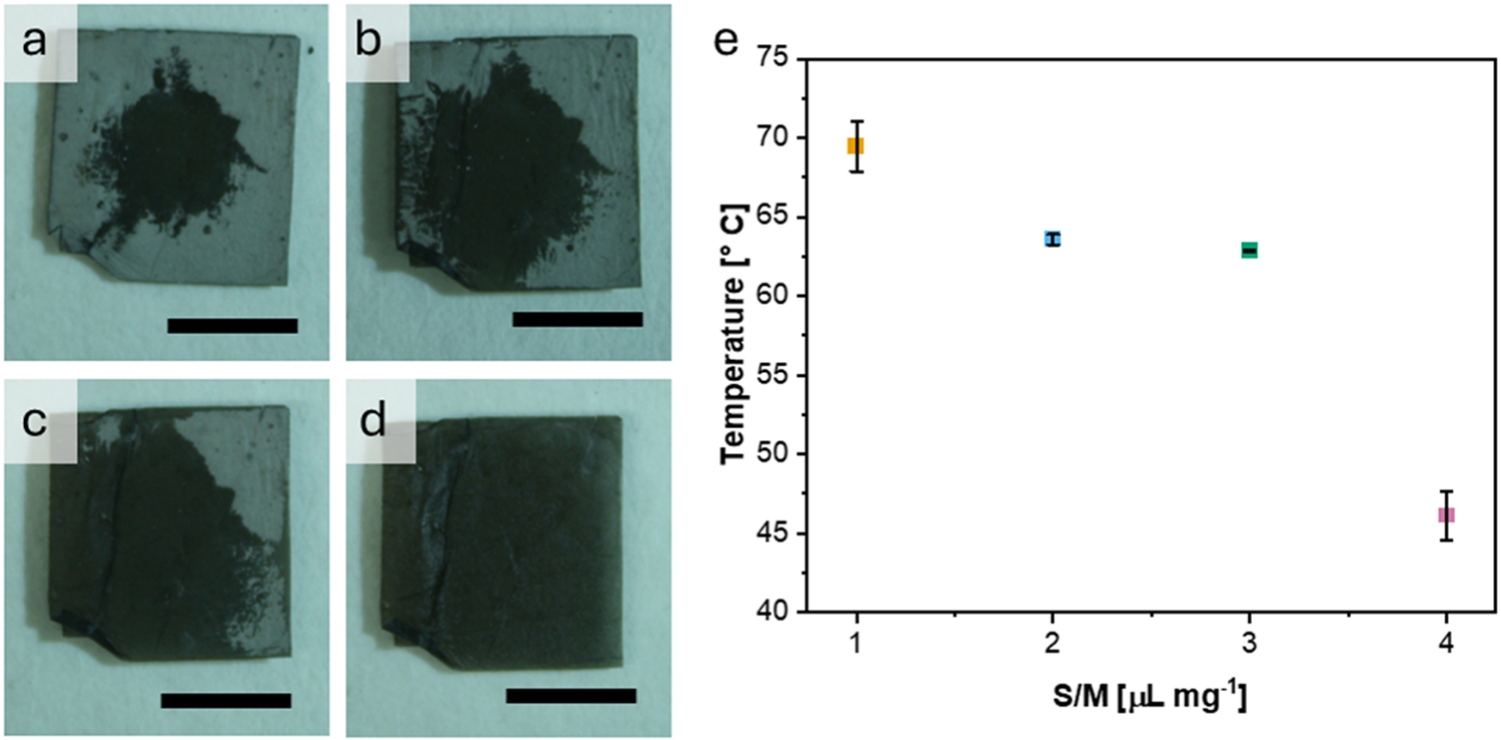
(a–d) Photographs of the mat impregnated with GVL solvent at different *S*/*M* ratios: (a) 1 μL mg^–1^, (b) 2 μL mg^–1^, (c) 3 μL mg^–1^ and (d) 4 μL mg^–1^ (scale bar: 5 mm). (e) Average *T* reached during 1 min of irradiation at 648 W m^–2^ with different amounts of GVL solvent.

Right after the addition of GVL, it was observed that in a mat of 1 × 1 cm^2^ with 1 μL mg^–1^ *S*/*M* (Figure 1a) and 2 μL mg^–1^ *S*/*M* (Figure 1b), the solvent was confined to a spot with a diameter of 4.8 ± 0.4 mm and 6.9 ± 0.3 mm, respectively. By reaching 3 μL mg^–1^ *S*/*M*, the solvent spread beyond a circular spot, covering nearly half of the electrospun surface (Figure 1c). The entire surface became saturated at 4 μL mg^–1^ *S*/*M* (Figure 1d). As shown in Figures 1e and S1, the temperature of the electrospun nonwoven irradiated with the LED light decreases with the quantity of solvent used, thus confirming the heat-dissipative effect of the solvent. In particular, with 1 μL mg^–1^ *S*/*M*, the sample temperature reached almost 70 °C. The temperature was approximately 63 °C with 2 μL mg^–1^ and 3 μL mg^–1^, and only 45 °C with 4 μL mg^–1^. These initial results prompted us to select 2 μL mg^–1^ as the optimal option for the following experiments, as the solvent spot was as large as the irradiation spot (i.e., ø 7 mm), with limited heat dissipation.

Once the solvent quantity was determined, we evaluated the photothermal efficiency of the nonwovens in the removal conditions. Photothermal efficiency (η) is usually calculated for samples in solution^14^ but here we adapted the definition of η to our specific case (eqs S1–S4), reported in eq 1.

1 $$\eta =\frac{\mathrm{hS}\left(T_{\max}-T_{0}\right)}{I}$$

Here, *T*_max_ and *T*_0_ represent respectively the maximum temperature reached during irradiation and the ambient temperature, and I represents the irradiation power. *h* and *S* are respectively the heat transfer coefficient and the surface area of the sample; the term *hS* was found fitting the heating curve of the photothermal nonwoven during irradiation (Figure S2). The photothermal efficiency for the PULL_M nonwovens in the presence of GVL 2 μL·mg^–1^ was found to be ∼70%, which is very close to that of the sepia melanin NPs dispersion in water (68%),^41^ denoting that the NPs preserve their photothermal properties even when embedded within the fibers of the nonwoven.

Another critical aspect to consider when using photothermal nonwovens is the actual temperature at the interface between the mat and the surface to be cleaned. In our previous work, we monitored the temperature of both the front and back of the nonwoven during LED irradiation of the front side, both in dry conditions and in the presence of the solvent. In the absence of solvent, a significant temperature gradient was found between the two sides of the mat. Conversely, in the presence of the solvent, no appreciable temperature gradient was detected, owing to the higher thermal conductivity of GVL; thus, the temperatures of the front and back sides of the mat were equal. During the application, the irradiated mat loaded with the solvent is in direct contact with the substrate to be cleaned, which may contribute to heat dissipation. In the previous study,^5^ the temperature at the mat–substrate interface was not directly measured, as the primary objective was to demonstrate the feasibility of the photothermal approach rather than to tailor selective layer removal. In the present work, accurate knowledge of the interfacial temperature is essential to design treatment conditions that enable selective removal of the external coating while preserving the underlying polymer matrix. To investigate the temperature that is observed on the mock-up surface, a thermocouple was placed on the top of it, in direct contact with the AlkB surface to be removed, beneath the GVL-loaded electrospun mat, as shown in Figure 2a.

**Figure 2:**
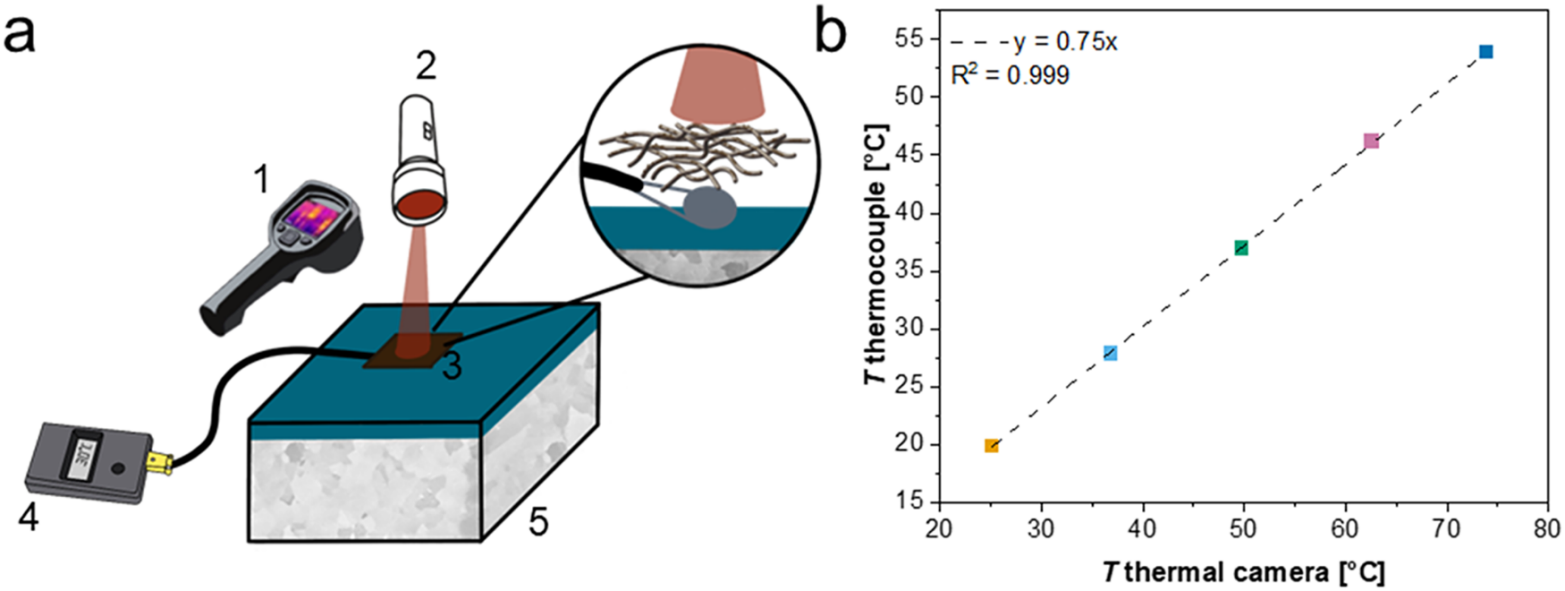
(a) Scheme of the setup employed for monitoring both the temperature of the irradiated electrospun mat and that at the mock-up surface (1: thermal camera; 2: LED; 3: electrospun mat with the irradiated spot; 4: thermocouple; 5: mock-up). (b) Linear correlation between the temperature registered on the top of the PULL_M mat with the thermal camera and the temperature registered in contact with the painted mock-up with the thermocouple, at different LED irradiances.

Figure S3 reports the observed temperature difference (Δ*T*) on the top and on the back of the mat applied on the mock-up surface as a function of the LED irradiance. As expected, the temperature recorded by the thermal camera is always higher than the one measured with the thermocouple. This confirms the hypothesis that the surface of the substrate efficiently dissipates heat. Not surprisingly, Δ*T* increases with irradiance, as the temperature rise at the exposed surface is greater than at the interface with the substrate, the latter being influenced by the substrate’s thermal diffusivity. The linearity between the temperature registered on top of the mat and the one in contact with the painting surface (Figure 2b) allows defining appropriate irradiation conditions for the removal procedure. Moreover, to select the most appropriate removal conditions, the glass transition temperatures (*T*_g_) of both the alkyd and acrylic varnishes were determined (Figure S4). This parameter is of high relevance since when polymers are heated above their *T*_g_, the macromolecular chains gain rotational freedom, with a massive increase of free volume, which can better accommodate molecules of solvents. As a consequence, the varnish solubility (or swelling in the case of cross-linked polymers) is favored at temperatures higher than the polymer *T*_g_. The *T*_g_ of both the blue and yellow cross-linked alkyd varnishes is about 22 °C, while the *T*_g_ of the yellow acrylic varnish is about 58 °C. Based on the linear correlation reported in Figure 2b, to perform removal experiments, we have selected two different irradiances, named “low” and “high”. These conditions allowed achieving, respectively, 28 and 56 °C at the mat surface (as measured by the thermal camera), which correspond to approximately 22 and 41 °C at the mat/mock-up interface (based on the linear correlation reported in Figure 2b). Therefore, at “low irradiance” the removal test is carried out at a temperature close to the *T*_g_ of the alkyd layer, while at “high irradiance” the temperature is largely above. In both cases, the removal temperature is below the *T*_g_ of the acrylic varnish.

### 3.2 Evaluation of the Removal Performance

Two representative multilayer polymeric systems were recreated at laboratory scale to model situations in which an external cross-linked coating must be selectively removed without compromising an underlying polymer layer (Figure S5). In the first scenario, a substrate was coated with a yellow acrylic varnish (AcrY), representing a solvent-sensitive polymer layer, and then overlaid with a blue cross-linked alkyd varnish (AlkB), simulating an insoluble external coating (AlkB on AcrY mock-up). In the second scenario, the underlying layer consisted of a yellow alkyd varnish (AlkY), resulting in a chemically similar alkyd-on-alkyd multilayer system (AlkB on AlkY mock-up).

In both cases, the key challenge is the complete removal of the undesired cross-linked AlkB layer while preserving the integrity of the polymeric layer beneath. The first configuration represents the most demanding scenario, as the solvent required to swell the cross-linked alkyd coating may also interact with and partially dissolve the underlying un-cross-linked acrylic layer, making selective removal particularly challenging. Such multilayer architectures are common in protective and decorative coating systems, where an external cross-linked topcoat must be removed or reworked without affecting the functional underlayer. They are also typically encountered when varnished from vandalic acts need to be removed from urban art or, more generally, urban furniture.

The removal treatments were performed using the following conditions: (i) electrospun mat fully saturated with GVL and applied for 5 min without irradiation; (ii) electrospun mat loaded with 2 μL mg^–1^ S/M and applied for 2.5 min with low irradiance (*T*_thermalcamera_ = 28 °C); (iii) same conditions of (ii) with high irradiance (*T*_thermalcamera_ = 56 °C); (iv) same conditions of (ii) and (iii) without irradiation. After the application of the electrospun fibers, cotton swabs were always used to remove the swelled AlkB layer. A multianalytical protocol was used to investigate two crucial removal aspects: residual unwanted material on the treated surface and the method invasiveness.

#### 3.2.1 Evaluation of the Removal Efficacy

Three-dimensional images via Hirox digital microscopy were acquired to evaluate the surface topography after the different removal treatments. Images were initially acquired from areas of the yellow varnish layer, serving as references. They show a heterogeneous surface appearance, due to depressions attributable to the porosity of the cementitious mortar substrate, which creates holes in the surface (Figure 3a-x,3b-x).

**Figure 3:**
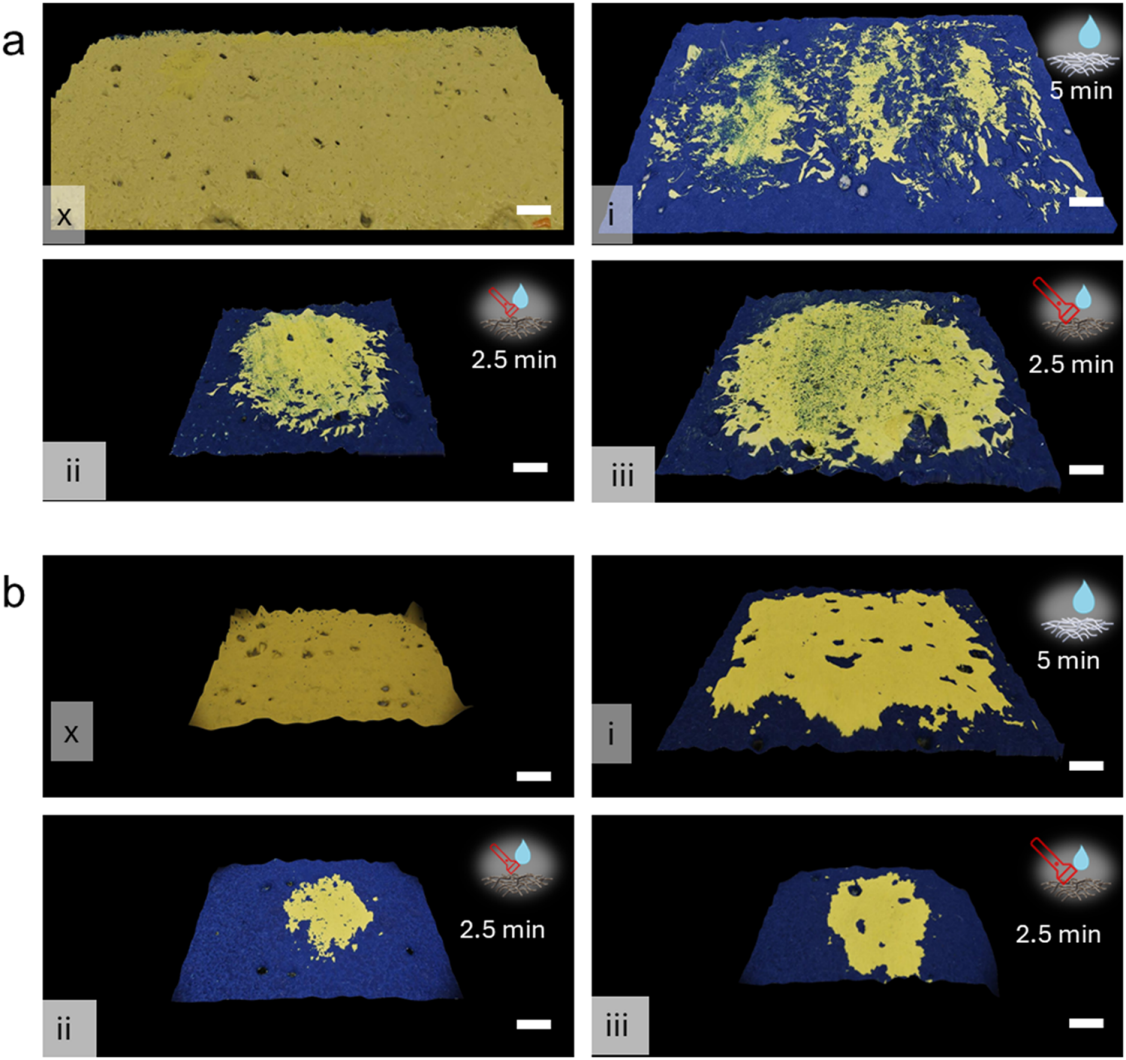
3D micrographs of (a) AlkB on AcrY mock-up and (b) AlkB on AlkY mock-up: (x) the AcrY or AlkY reference area, and (i–iii) the areas cleaned by using (i) electrospun mat fully saturated with GVL and applied for 5 min without irradiation; (ii) electrospun mat loaded with 2 μL mg^–1^ *S*/*M* and applied for 2.5 min with low irradiance (*T*_thermalcamera_ = 28 °C); (iii) same conditions of (ii) with high irradiance (*T*_thermalcamera_ = 56 °C). Scale bar: 1 mm.

On the AlkB on AcrY mock-up, the photothermal treatment (Figure 3a-ii–iii) proved to be necessary to remove the AlkB layer, as without irradiation, the AlkB could not be eliminated (Figure S6a). Even the increase of both GVL amount and the treatment time did not allow a complete removal of the blue alkyd varnish (Figure 3a-i). Notably, a visible dragging and mixing of the blue paint into the underlying yellow layer (Figure 3a-ii–iii) can be observed. This phenomenon could be linked to a partial solubility of the underlying acrylic layer in the presence of the GVL, which may have favored the mixing of the two layers, with a consequent visual effect of dragging. In the case of AlkB on AlkY, the removal treatments using the fully saturated mat (Figure 3b-i), as well as the treatments with half the GVL amount combined with irradiation (Figure 3b-ii–iii), were effective in removing the AlkB. These results demonstrate the effectiveness of the photothermal treatment and its significant contribution to enhancing the removal efficacy. Indeed, the use of half the solvent alone without irradiation could not remove the AlkB (Figure S6b).

Interestingly, increasing the solvent loading to the saturation level without irradiation led to different removal results, depending on the nature of the layer below. In particular, the removal of AlkB remains incomplete and heterogeneous if the underlying layer is acrylic (AlkB on AcrY, Figure 3a-i). Conversely, the process is notably more effective when the underlying layer is alkyd-based (Figure 3b-i). This discrepancy suggests a different interaction/adhesion between the blue alkyd varnish and the underlying binder, potentially influencing the dissolution and the removal mechanisms.

Hyperspectral imaging in the visible–near-infrared range was employed to evaluate the results and compare the amount of the residual blue paint in the treated areas. Principal Component Analysis (PCA) was applied to the spectral signatures obtained from the hyperspectral image to reduce data dimensionality and enhance the interpretability of spectral variance.^42^ With 96.41% of the total variance explained, PC1 captures nearly all meaningful spectral variation mainly related to the reflection bands at 508 nm (yellow paint) and 760 nm (blue paint), as the corresponding loadings suggest (Figures S7a and S8a). This made PC1 the optimal single component to perform data segmentation by clustering it into three spatial clusters on the basis of the frequency distribution of PC1 score values. Threshold values for segmentation were determined by identifying local minima and inflection points in the histogram of PC1 score values (Figures S7b and S8b). The defined thresholds were used to construct a false-color RGB map: pixels with high PC1 scores (>0.047) appear in red, those with intermediate scores (−0.028 to 0.047) in green, and those with low scores (<−0.028) in blue (Figure S7c and S8c). Finally, the average reflectance spectra were extracted from each of the segmented clusters (Figure 4b,d). The average spectrum from the red pixel clusters is associated with the yellow reference, which is characterized by a single peak in first derivative at 508 nm. The blue pixels reflected the spectral profile of the blue paint, with spectra characterized by a peak at 760 nm and a less intense peak at around 650 nm in first derivative. Green pixels presenting the signal of both the yellow and the blue pigment corresponded to the green halo, which was ascribed to the dragging and mixing of the blue varnish onto the yellow one in the AlkB on AcrY mock-up (Figure 4a). Thus, counting the number of pixels in the RGB image allows for an estimation of the cleaned, partially cleaned, and uncleaned areas. It is worth noting that the aim of this elaboration is not to provide an absolute quantification, but rather to propose a semiquantitative approach for comparing the different removal systems.

**Figure 4:**
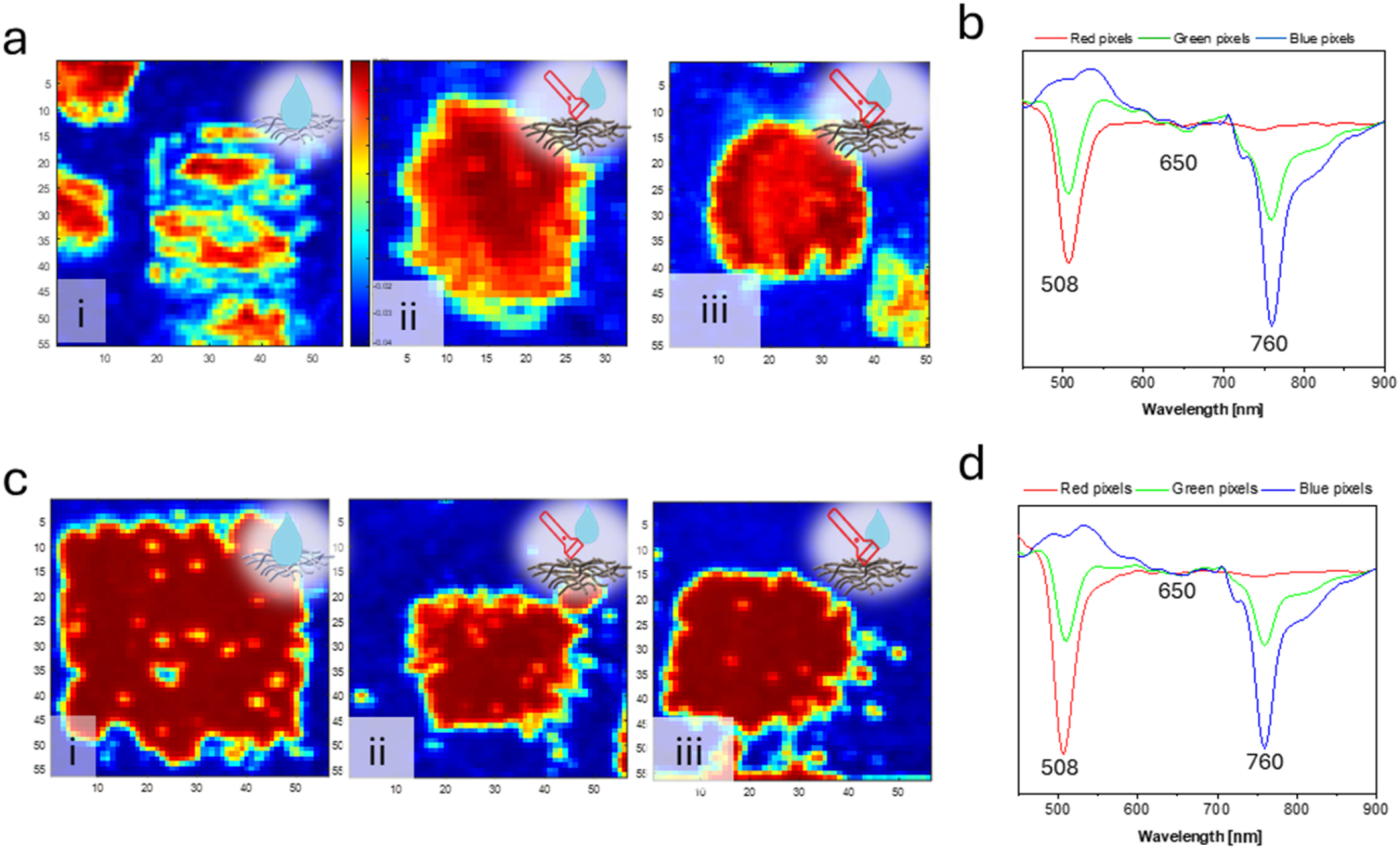
False color PC1 score map of the representative areas of AlkB on AcrY (a) and AlkB on AlkY (c) after removal by using (i) electrospun mat fully saturated with GVL and applied for 5 min without irradiation; (ii) electrospun mat loaded with 2 μL mg^–1^ *S*/*M* and applied for 2.5 min with low irradiance (*T*_thermalcamera_ = 28 °C); (iii) same conditions of (ii) with high irradiance (*T*_thermalcamera_ = 56 °C). (b, d) Average reflectance spectra extracted from each of the segmented clusters.

In the AlkB on AcrY mock-up, the use of GVL-loaded pullulan at room temperature could not ensure a proper removal, showing the persistence of relevant (>20%) uncleaned areas (Table 1, entries 1 and 2). On the contrary, the photothermal action allowed for the reduction of the unclean area to 0%, both at high and low irradiance (Table 1, entries 3–6). By comparing the two irradiance conditions, it can be observed that increasing the temperature does not significantly improve removal efficacy, while greater variability in cleaning effects is observed, suggesting a possible reduction in treatment reproducibility and a slight increase in invasiveness. Overall, milder conditions appear to be sufficient to promote the removal of the external alkyd layer.

**Table 1:** Results of the Percentage Quantification of Red, Green, and Blue Pixels of the Hyperspectral Images That Are Associated with the Clean, Partially, and Uncleaned Parts of the AlkB on AcrY Mock-Up Areas Treated with Three Different Removal Conditions

Entry	Retentive system	*S*/*M* [μL mg^–1^]	Time of application [min]	*T*_thermalcamera_ [° C]	Cleaned area [%]	Partially cleaned area [%]	Uncleaned area [%]
1	PULL	4.5	5	rT	6	64	30
2	PULL	4.5	5	rT	3	76	21
3	PULL_M	2	2.5	28 ± 2	67	33	0
4	PULL_M	2	2.5	28 ± 2	65	35	0
5	PULL_M	2	2.5	56 ± 2	45	55	0
6	PULL_M	2	2.5	56 ± 2	71	29	0

In the AlkB on AlkY mock-up, visual inspection did not reveal significant differences after applying the various removal methods. As the spatial resolution of the hyperspectral camera is only 300 × 300 μm^2^, quantifying the relative coverage of the clean, uncleaned and partially cleaned zones on the treated areas is unlikely to yield more insights than visual observations. For this kind of sample, it can be noted that the green pixels can also be found, but only at the edges of the cleaned areas (Figure 4b), where the thinning of the blue varnish revealed the spectral features of the underlying yellow layer.

#### 3.2.2 Removal Selectivity and Invasiveness

Removal efficacy is not the only factor to consider during removal as selectivity toward the targeted layer is equally crucial. In other words, the unwanted layer should be removed as thoroughly as possible, while the underlying layer should be preserved in its original state. The potential removal of the yellow layer during the various removal treatments was assessed by liquid chromatography with diode array detection (HPLC-DAD). To this aim, the yellow dye (i.e., PY74) extracted from the cotton swabs used for removal was quantified through external calibration (Figure S9). No traces of PY74 were detected in the cotton swabs after removal the AlkB on AlkY mock-up, meaning that the cross-linked alkyd yellow varnish well-resists all the tested removal methods.

Conversely, in the case of AlkB on AcrY, a partial removal of the layer below occurs since the chromatograms of the cotton swabs used to clean this mock-up show a peak at around 24.6 min, which is attributed to PY74 (Figure S10). In Table 2, the PY74 quantification, normalized by the treated area, shows that using the mats fully saturated with the solvent not only leads to an unsatisfactory removal (Figure 4a-i) but also to the removal of a high amount of yellow dye in the area where the removal is more effective (0.77 μg mm^–2^ Table 2, entry 1). At low irradiance, the removal treatment appears more selective, as the amount of PY74 removed per unit area ranges between 0.05 and 0.2 μg mm^–2^ (Table 2 entries 3 and 4). In contrast, higher-temperature treatment results in greater material removal (approximately 0.35 μg mm^–2^ PY74 removed for unit area, Table 2 entries 5 and 6). These results highlight that high removal temperatures should be avoided to limit the solubilization of the acrylic layer to be preserved.

**Table 2:** Results of the HPLC-DAD Quantification of PY74 Detected on the Cotton Swabs Used for Removal under Various Conditions, the AlkB on AcrY Mock-Up (rT = Room Temperature)

Entry	Retentive system	*S*/*M* [μL mg^–1^]	Time of application [min]	*T*_thermalcamera_ [° C]	PY74 per unit area [μg mm^–2^]
1	PULL	4.5	5	rT	0.77
2	PULL	4.5	5	rT	0.15
3	PULL_M	2	2.5	28 ± 2	0.20
4	PULL_M	2	2.5	28 ± 2	0.05
5	PULL_M	2	2.5	56 ± 2	0.35
6	PULL_M	2	2.5	56 ± 2	0.36

The assessment of the preservation state of the underlying layers was carried out to provide a complementary evaluation of the selectivity of the overpaint removal. This included monitoring both the chemical and mechanical condition of the layers to be preserved using ATR-mode infrared microspectroscopy and Brillouin (BLS) microspectroscopy, respectively.

Infrared spectra were acquired from both the yellow paint reference and the areas treated with the various removal methods. As no significant spectral differences were observed among the different treatments, only the spectrum corresponding to the area treated under the highest temperature conditions (i.e., high irradiance; *T*_thermalcamera_ = 56 °C) is shown for both mock-ups (Figure 5).

**Figure 5:**
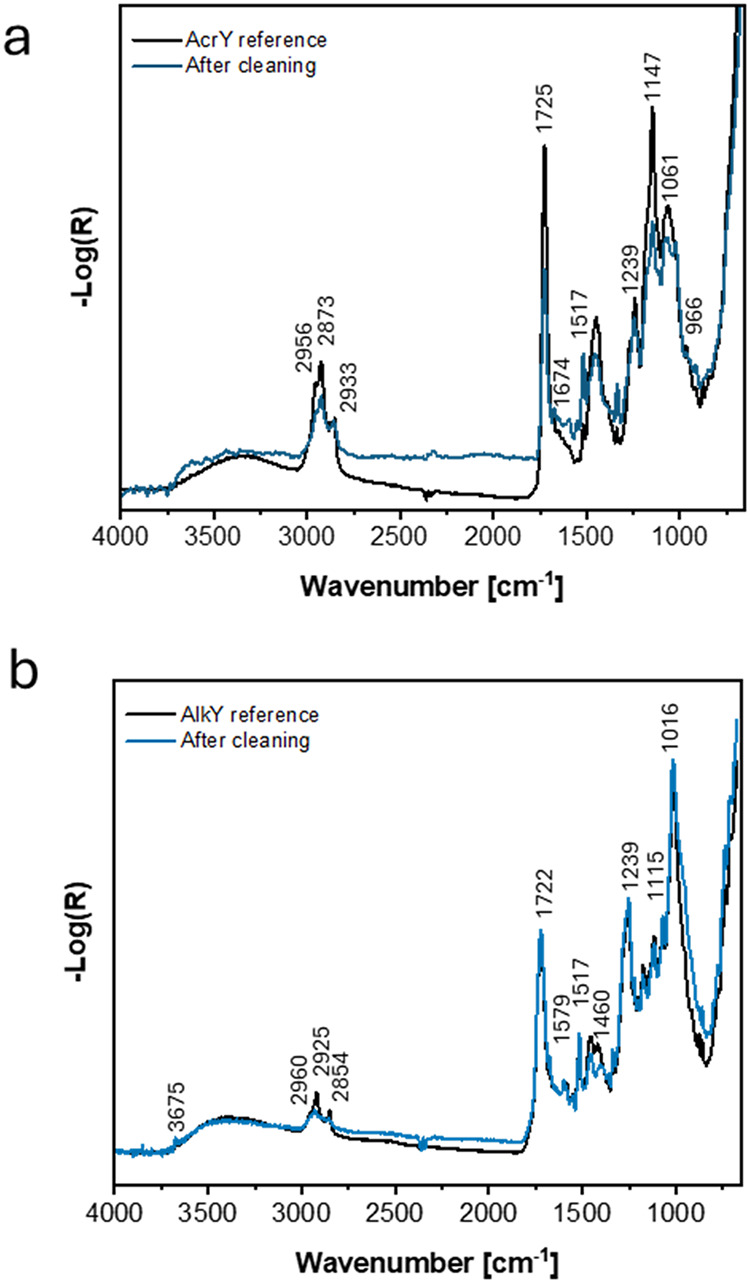
(a) μATR-FTIR spectra of AlkB on AcrY mock-up after removal (blue) and the corresponding AcrY reference (black); (b) μATR-FTIR spectra of AlkB on AlkY mock-up after removal (blue) and the corresponding AlkY reference (black). The spectra after removal were acquired on the areas treated with the electrospun mat loaded with 2 μL mg^–1^ *S*/*M* and applied for 2.5 min with high irradiance.

Both the AlkB on AcrY spectra collected after removal and the reference AcrY (Figure 5a) exhibited distinctive peaks corresponding to the acrylic binder (2956, 2933, 2873 cm^–1^ C–H stretching, 1725 cm^–1^ ester C=O stretching, 1485 cm^–1^ C–H bending, 1269, 1239, 1147, 1061, 966 cm^–1^ C–O and C–C stretching), as well as those attributed to the yellow pigment PY74 (1674, 1593, 1554, 1519, 1403, 1362, 1339, 1297, 1177, 1085, 1044, 916, 800 cm^–1^),^43,44^ Similarly, the characteristic peaks of the alkyd varnish appear in the spectra of both AlkB on AlkY after removal and AlkY reference (Figure 5b): 2960, 2925, 2854 cm^–1^ C–H stretching, 1722 cm^–1^ ester C=O stretching, 1597, 1577 cm^–1^ aromatic skeletal ring vibrations, 1460 cm^–1^ C–H bending, 1115, 1070 cm^–1^ C–O and C–C stretching. Moreover, both spectra display the previously mentioned peaks corresponding to the yellow pigment PY74.^20,43,44^ Other peaks observed in all spectra are ascribed to titanium dioxide (PW6) (from 750 to 650 cm^–1^) and talc (3676 and 1015 cm^–1^).^44^ In brief, infrared spectroscopy demonstrates that after removal, the yellow varnishes have preserved their chemical structure, suggesting that the photothermal treatment maintains the molecular integrity of the substrate, further supporting its suitability for conservation applications.

Brillouin light scattering is a powerful technique for studying viscoelastic properties of materials in a noninvasive way and has only recently been applied in the field of heritage science.^39,45^ It deals with the interaction of the incident photon with thermally activated acoustic waves (phonons) propagating in the material. Specifically, the Brillouin frequency shift can be used to calculate the material’s longitudinal elastic modulus (*M*), considering its density and refractive index. On the other hand, the full width at half height is related to the acoustic attenuation, and therefore to the material’s viscoelastic properties. Figure 6a compares the average BLS spectra of point measurements collected from the AlkY, AcrY, and AlkB layers. The comparison highlights how the mechanical properties of the final films depend both on the type of varnish—acrylic or alkyd—and on the presence of yellow or blue pigments. In detail, for the same pigment, the BLS peak of alkyd yellow appears at a higher frequency (14.72 GHz) than that of acrylic yellow (14.12 GHz), indicating greater stiffness in the acrylic-based film. This result is consistent with the higher *T*_*g*_ measured for the acrylic paint compared with the alkyd one. Conversely, when comparing different pigments within the same binder, the alkyd blue shows a peak at a lower frequency (10.64 GHz) than alkyd yellow, suggesting a softening effect. Notably, the influence of pigments on the mechanical properties of the final film has also been observed in previous studies using linseed oil as a binder.^39^ Figure 6b,c show the average BLS spectra, normalized and vertically shifted, of the punctual measurements collected from the AcrY and AlkY areas after the different removal treatments: (i) electrospun mat fully saturated with GVL and applied for 5 min without irradiation (r*T*-orange); (ii) electrospun mat loaded with 2 μL mg^–1^ *S*/*M* and applied for 2.5 min with low irradiance (Low *T*, pink); same conditions of (ii) with high irradiance (High *T*, green). For visualization purposes, only the anti-Stokes side of the Brillouin spectra is presented in Figure 6. This allows a more precise comparison of the frequency shifts across the different samples. However, the full Brillouin spectrum typically includes a central elastic peak at 0 GHz, along with symmetric Stokes and anti-Stokes peaks. The average frequency shifts obtained from the fitting procedure are shown in Table 3 for all the removal procedures along with the references. From the results obtained, it is possible to observe that in both samples, AlkB on AcrY and AlkB on AlkY, independently from the removal procedure employed, the elastic properties of the layer to be preserved, expressed by the Brillouin frequency shift, are unchanged when compared to the reference. These results lead to the conclusion that the removal methods proposed here do not alter the mechanical properties of the varnish layer to be preserved.

**Figure 6:**
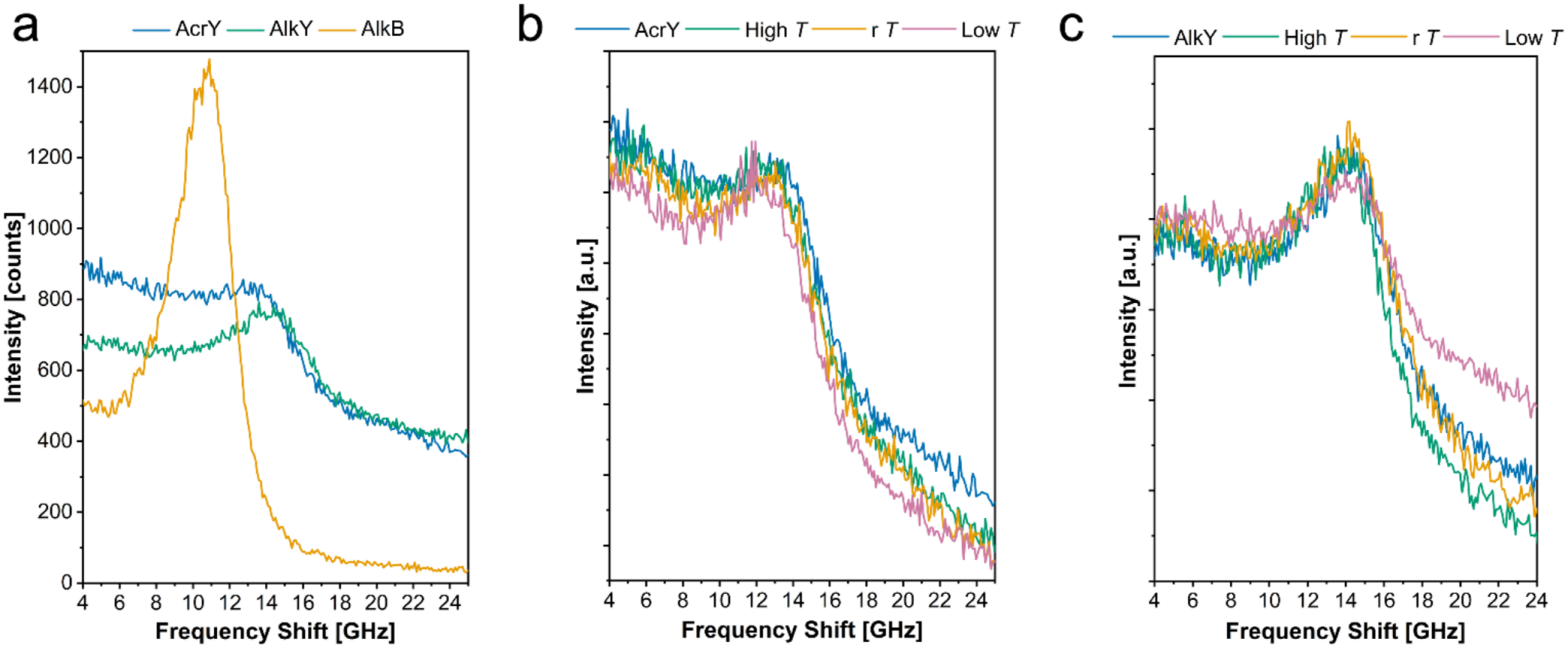
(a) Average BLS spectra obtained for the AcrY (blue line), AlkY (green line) and AlkB (orange line) references; Average BLS spectra, normalized and vertically shifted, of the two mock-ups AlkB on AcrY (b) and AlkB on AlkY (c) after removal with (i) electrospun mat fully saturated with GVL and applied for 5 min without irradiation (r*T*-orange); (ii) electrospun mat loaded with 2 μL mg^–1^ *S*/*M* and applied for 2.5 min with low irradiance (Low *T*, pink); same conditions of (ii) with high irradiance (High *T*, green), along with the AcrY and AlkY reference spectra (AcrY and AlkY, blue). A minimum of three measurements was taken for each sample. For visualization purposes, only the anti-Stokes side of the Brillouin spectra is shown.

**Table 3:** Results of the Brillouin Peak Fitting on the AcrY, AlkY, and AlkB Paint References and the Cleaned Areas of AlkB on AcrY and AlkB on AlkY Mock-Ups

Reference sample	Brillouin Frequency Shift [GHz]
AcrY	14.12 ± 0.02
AlkY	14.72 ± 0.03
AlkB	10.64 ± 0.03

Removal treatment	Brillouin Frequency Shift [GHz]	
	AlkB on AcrY	AlkB on AlkY
*rT*	14.12 ± 0.03	14.72 ± 0.03
Low*T*	14.12 ± 0.04	14.72 ± 0.03
High*T*	14.12 ± 0.03	14.72 ± 0.04

### 3.3 Reusability of the Photothermal Electrospun Mats

The first principle of green chemistry emphasizes waste reduction.^46–48^ To align with this objective, we investigated the potential for reusing the same electrospun mats across multiple removal applications. Specifically, we examined the preservation of fibrous morphology and photothermal properties after 3, 5, and 10 use cycles employing the milder temperature conditions described above (low irradiance), which were the best conditions to obtain acceptable removal performances and the best selectivity. As shown in Figure 7a, no evident differences can be detected by SEM in the fibrous morphology between the preuse mat (Figure 7a-i) and those after 3 (ii), 5 (iii), and 10 (iv) removal applications, a finding further supported by the unmodified average fiber diameter of each mat (0.46 ± 0.05 μm, 0.43 ± 0.07 μm, 0.48 ± 0.06 μm, 0.43 ± 0.08 μm, respectively before use, and after 3, 5, and 10 removal treatments). Additionally, upon irradiation, the average *T* did not significantly change after 3, 5, and 10 removal treatments, suggesting that the photothermal properties are maintained (Figure 7b). From a macroscopic perspective, a slight loss of cohesion in the outermost fibers due to handling was noted in the mats after three reuses (Figure S11). Therefore, while the material can be reused for at least 10 cycles, three cycles were identified as an optimal reuse threshold. This observation was verified by performing mechanical tests under tensile mode that demonstrated that the practical handling robustness of the material is preserved after up to three reuses (Figure S12 and Table S1). More specifically, the mat after three reuses experienced a compaction of the layers that induced a thickness reduction of ca. 25%. A comparable thickness reduction was also observed when the nonwoven was immersed in GVL and dried without any irradiation treatment or usage, demonstrating that the compaction phenomenon is associated with solvent exposure itself rather than with the photothermal treatment process and its use. Giving the mat compaction and the consequent decrease of porosity which renders a direct comparison of mechanical properties in terms of stress unreliable, mechanical data were compared in terms of force–strain analysis, which demonstrated that, under the same applied force, the pristine and reused mats undergo comparable deformation, indicating that the practical mechanical robustness of the material is preserved after three reuse cycles despite the partial densification of the structure. Importantly, the removal efficacy of an AlkB from AlkY remained consistent across three removal applications. Indeed, as shown in Figure 7c, the treated areas maintained similar dimensions, and the only blue varnish residues are confined in the porosities of the substrate, in accordance with what was observed in all the other tests performed on the AlkB on AlkY sample.

**Figure 7:**
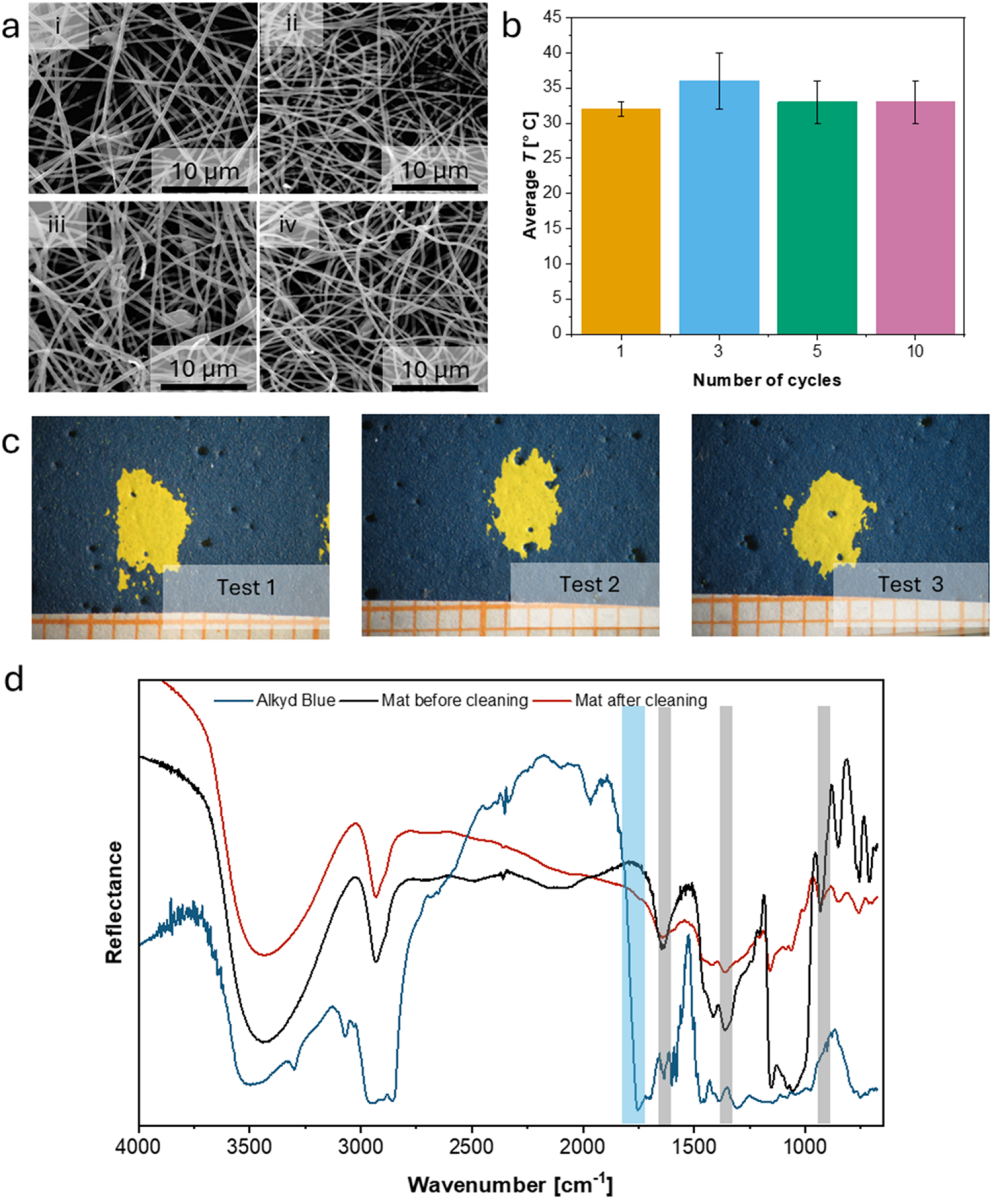
(a) SEM micrographs of the mats before use (i), and after being used for removal 3 times (ii), 5 times (iii) and 10 times (iv). (b) Average *T* reached at the 1st, 3rd, 5th, and 10th removal applications. (c) Areas of the mock-up cleaned with the same electrospun mat used for three consecutive removals (low irradiance). (d) Spectra in total reflectance of the alkyd blue spray paint (blue) and the mat before use (black); average spectra of the total reflectance map acquired on the mat after 3 uses (red). Characteristic peaks are highlighted.

An FTIR microscopic map in reflection mode was performed on the electrospun mats after removal to highlight the presence of possible AlkB residues on the mat surface. Figure 7d compares the reference spectra of the paint to be removed and the precleaned mat with the average spectra extracted from the FTIR map acquired on the mat after three removal applications. The latter exclusively exhibited the spectral profile of the electrospun mat, characterized by distinctive pullulan peaks (1640 cm^–1^ C–O–C glycosidic bridge, 1363 cm^–1^ C–OH bending, 929 cm^–1^ α (1–6) and α (1–4) linkage).^49–51^ Additionally, the CH stretching bands present a highly different shape from the ones of the alkyd paint, and the characteristic peak of the C=O bond at 1755 cm^–1^ is absent.^52,53^ This demonstrates the absence of blue alkyd residues in the mats after three removal applications, confirming their reusability.

## 4 Conclusion

In this study, we present a reusable photothermal electrospun nonwoven based on pullulan fibers incorporating melanin nanoparticles for the selective removal of polymeric surface coatings. The material combines the high surface area of electrospun nonwovens with the efficient light-to-heat conversion capability of melanin, enabling localized swelling at polymer–polymer interfaces.

Two representative multilayer polymer systems were investigated, consisting of cross-linked alkyd coatings applied onto either an acrylic or an alkyd substrate. By tailoring solvent loading and irradiation conditions according to the glass transition temperature of the involved polymer layers, selective removal of the external alkyd insoluble coating was achieved. A custom-designed setup allowed measurement of the temperature achieved by the irradiated mat, both on its outer surface and at the interface with the underlying substrate, verifying the linear correlation between the external temperature and the one measured at the specimen, thus facilitating the selection of specific irradiance levels for the removal treatment. The optimized photothermal approach reduced solvent consumption and application time compared to nonirradiated treatments. Multianalytical characterization confirmed high selectivity, with only trace removal of the underlying acrylic layer and no detectable alteration of the alkyd substrate. More specifically, the cleaning efficacy of the system depends on the nature of the underlying layer. In the AlkB on AcrY system, a dragging and mixing effect was observed, indicating localized superficial morphological modifications of the acrylic layer, likely associated with partial solvent interaction. Nevertheless, no changes in chemical composition or viscoelastic properties were detected, demonstrating that the bulk properties of the substrate remain unaffected. In this context, the outcome represents a significant improvement if compared with the ineffective results obtained using the solvent action without the photothermal treatment. The reusability of the electrospun mats and the use of nontoxic materials highlight the sustainability of the proposed platform. Although the present study was conducted on small-scale samples to ensure precise control of interfacial thermal effects, the approach is inherently modular and can be extended to larger surfaces. Indeed, electrospun mats can be fabricated in larger formats, high quantity of melanin NPs can be potentially obtained with our purification process, and the photothermal activation can be extended using scalable irradiation sources (e.g., LED arrays or scanning systems), following appropriate optimization of treatment time and operational workflows depending on the size and complexity of the surface.

Overall, this work establishes photothermal electrospun nonwovens as an effective and controlled strategy for the selective manipulation of multilayer polymeric coatings, offering a sustainable alternative to conventional stripping methods. Although the use of bioderived components, green solvents, and reusable materials supports the sustainability of the proposed approach, a quantitative life cycle assessment, including processing steps and comparison with conventional methods, is required for a complete evaluation and will be addressed in future work. This innovative system is a preliminary proof of concept of potential applicability after further development and scalability optimization for graffiti removal from urban art and more generally, urban furniture.

## Supplementary Material



## Notes

Additional results on the thermal camera and thermocouple analysis, fiber distribution of the mats employed for the reuse cycles, photographic documentation of the mat’s surface after reuse cycles, mechanical characterization, overview of all the removal tests performed, thermal characterization of the spray paints, chemometrics elaboration of the hyperspectral images, further details on the chromatographic analysis (PDF)
