# Supplementary material for: Reusable and Interface-Confined Photothermal Electrospun Nonwovens for the Selective Removal of Polymeric Coatings
Source: ACS Appl Mater Interfaces. 2026 Jun 24;18(26):37201–13. doi: 10.1021/acsami.6c04133 (PMC13352508; doi:10.1021/acsami.6c04133)
Supplement: Supplementary file 1 [file am6c04133_si_001.pdf]

Supporting information

Reusable and Interface-Confined Photothermal Electrospun Nonwovens for the Selective removal of Polymeric Coatings

Francesca Ramacciotti‡[1], Arianna Menichetti‡[2], Maddalena Redi[1], Emilio Catelli[1], Giulia Di Cara[1], Giorgia Sciutto[1], Maria Letizia Focarete[1,3], Ilaria Degano[4], Laura Cartechini[5], Francesca Rosi[5], Sara Mattana[5], Martina Alunni Cardinali[6], Marco Montalti[2,3], Chiara Gualandi[1,3,7], Silvia Prati[1]**

[1]Department of Chemistry "Giacomo Ciamician", University of Bologna, Bologna, 40129, Italy

[2]Department of Chemistry "Giacomo Ciamician", University of Bologna, Rimini, 47922, Italy

[3]Department of Chemistry “Giacomo Ciamician” and INSTM UdR of Bologna, University of Bologna, Bologna, 40129, Italy;

[4]Department of Chemistry and Industrial Chemistry, University of Pisa, Pisa, 56124, Italy

[5]National Research Council, Institute of Chemical Science and Technologies “G. Natta” (CNR-SCITEC), Perugia, 01623, Italy

[6]Department of Chemistry, Biology and Biotechnology, University of Perugia, Perugia, 01623 Italy

[7]Interdepartmental Center for Industrial Research on Advanced Applications in Mechanical Engineering and Materials Technology, CIRI-MAM, University of Bologna, Bologna, 40123 Italy

*Corresponding authors: s.prati@unibo.it, c.gualandi@unibo.it

1. Electrospun mat photothermal properties characterization

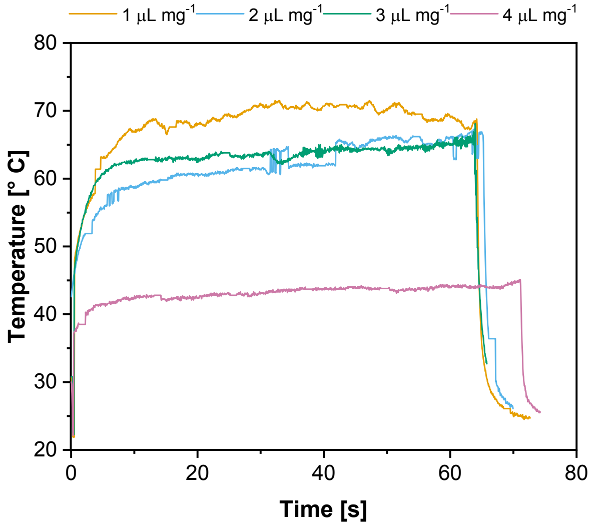

Figure S1. Representative measurements performed with the thermal camera on the electrospun mat impregnated with different *S/M* ratios: 1 μL mg^{-1} (yellow), c) 2 μL mg^{-1} (blue), d) 3 μL mg^{-1} (green) and e) 4 μL mg^{-1} (purple).

The photothermal efficiency (η) was obtained considering the expression used for samples in solution[1,2] that we adapted to characterize the photothermal nonwovens (Equation S1).

$$\eta = \frac{hS\,(T_{max} - T_0) - Q_D}{I(1 - 10^{-A_\lambda})} \qquad [S1]$$

Here, h and S are respectively the heat transfer coefficient and the surface area of the sample, and T_{max} and T_0 represent respectively the maximum temperature reached during irradiation and the ambient temperature. Q_D, which usually refers to the heat dissipated from the light absorbed by the cell and the solvent, in our case is the heat dissipated during light irradiation from the nonwoven in absence of melanin NPs. I and A_λ correspond to the irradiation light and the absorbance of the nonwoven at the irradiation wavelength, respectively.

Since the nonwoven without melanin (PULL) used as a reference did not display any temperature increase during irradiation, the Q_D term was measured to be 0 J·s^{-1}. Moreover, transmittance and reflectance measurements of the photothermal nonwovens (PULL_M) showed that the material has

a 0% transmittance and a 5% reflectance, thus it can be assumed that all the incident light is absorbed by the sample. As a result, the photothermal efficiency is calculated by Equation S2:

$$\eta = \frac{hS\ (T_{max} - T_0)}{I} \qquad [S2]$$

The term *hS* can be found considering the temperature variation during the heating process, which is represented by Equation S3[1]:

$$T - T_0 = (T_{max} - T_0)(1 - e^{-\frac{t}{\tau_s}}) \qquad [S3]$$

Where τ_s is a time constant for heat transfer that can be expressed as:

$$\tau_s = \frac{\sum_i m_i C_{Pi}}{hS} \qquad [S4]$$

In Equation S4, when the photothermal agent is dispersed in a solvent, *m* and C_P are respectively the mass and the specific heat capacity of the solvent. In our case, we considered the mass of the nonwoven and of the added GVL. As C_P, since within the irradiation area, the PULL_M is completely saturated by the solvent, we can assume that the prevalent heat capacity of the system is the one of GVL (1.8 $J \cdot g^{-1}\ °C^{-1}$)[3]. The term *hS* was thus found fitting the heating curve of the sample during irradiation (Figure S2).

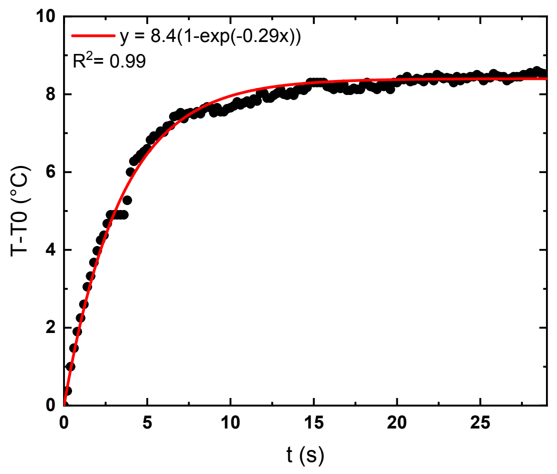

Figure S2. Example of temperature variation of PULL_M nonwoven in the presence of 2 $\mu L \cdot mg^{-1}$ GVL measured during irradiation at 660 nm. Fitting according to Equation S3 is plotted as a continuous red line.

Heating curves of four samples were analyzed: the value of hS was found at (0.0046 ± 0.0005) $J \cdot s^{-1}$ °C and the term T_{max}-T_0 at around (8.0 ± 0.4) °C. Given an irradiation power of 0.06 $J \cdot s^{-1}$, a photothermal efficiency of (70 ± 4) % resulted for the photothermal nonwovens.

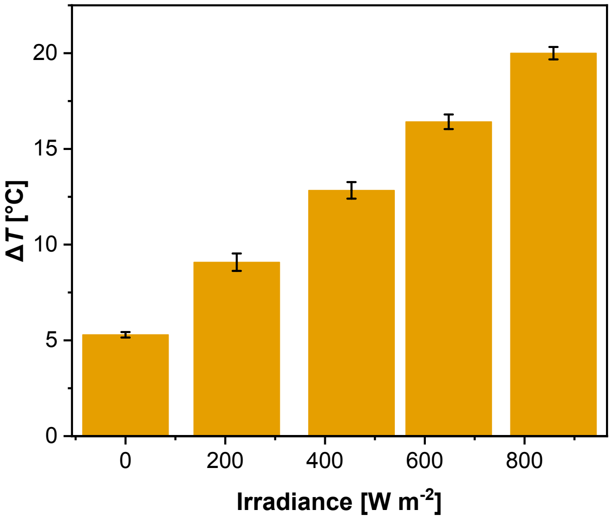

Figure S3. Temperature difference (ΔT) between the irradiated PULL_M mat side monitored with the thermal camera and the mock-up surface monitored with the thermocouple.

2. Evaluation of the removal performance

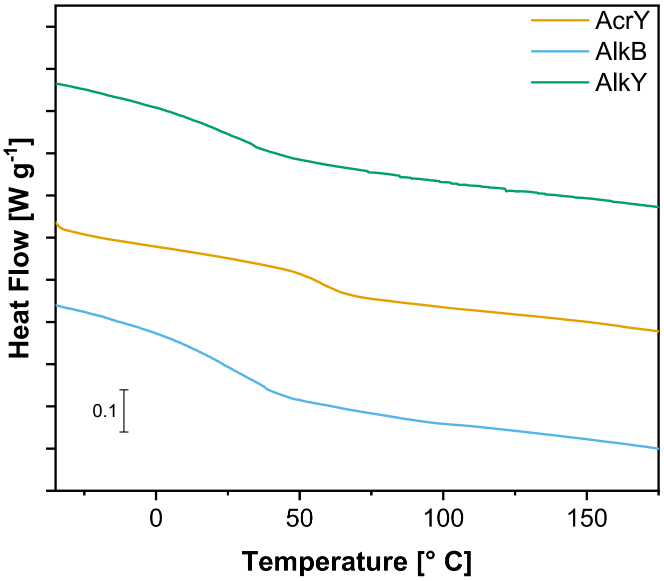

Figure S4. DSC curves of the three investigated varnishes: acrylic yellow (yellow), alkyd yellow (green) and alkyd blue (light blue).

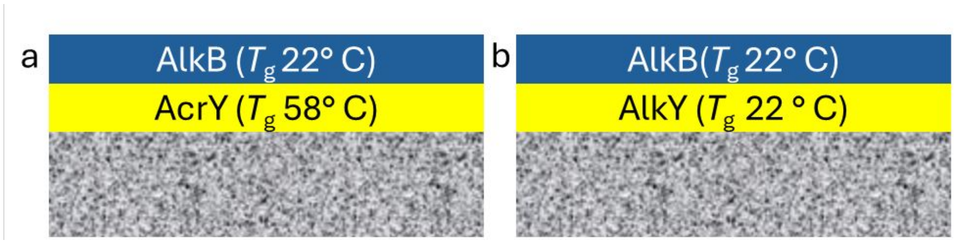

Figure S5. Schematic representation of the two model samples where the blue alkyd layer simulates the coating to be removed (e.g. a varnish from vandalic act), and the yellow layer simulate the polymer layer to be preserved (e.g. an artwork) realized with acrylic (a) and alkyd (b) spray paints. For each layer the glass transition temperature (T_g) is indicated.

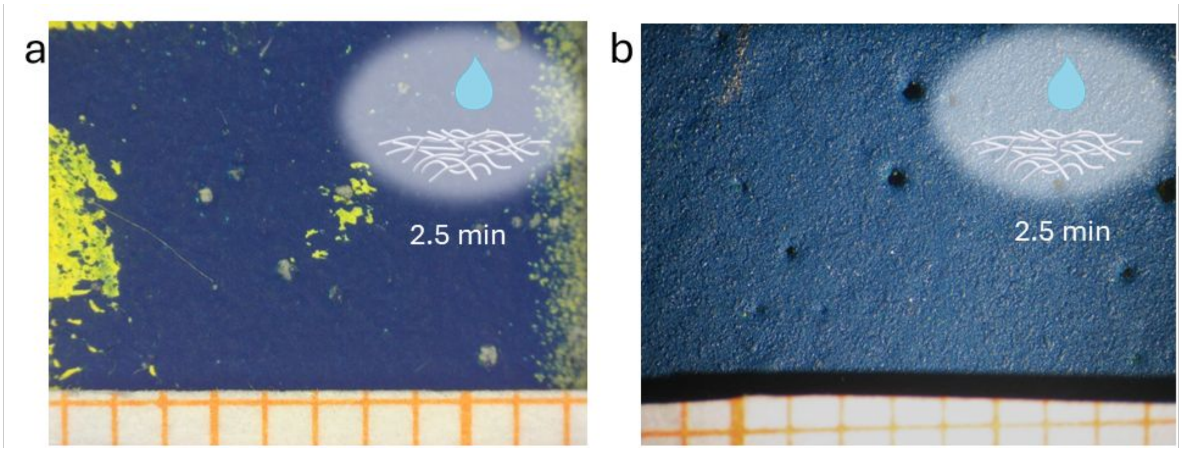

Figure S6. Representative pictures of AlkB on AcrY (a) and AlkB on AlkY (b) after removal procedure by using electrospun mat loaded with 2 μL mg^{-1} *S/M* and applied for 2.5 min without irradiation.

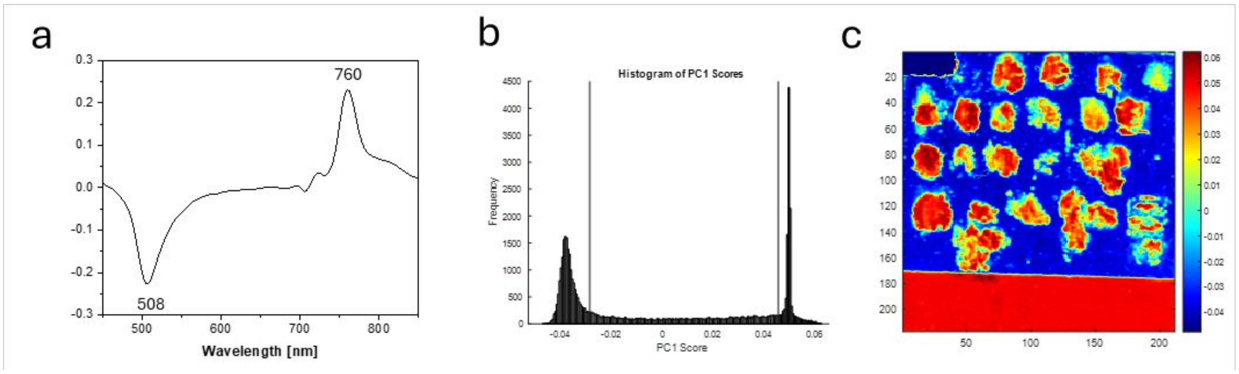

Figure S7. Results of PCA performed on the sample AlkB on AcrY. a) Loadings profile along PC1 b) Distribution of the score values along PC1, with identification of the thresholds (black lines) selected for the segmentation of the RGB score map. c) False color PC1 score map.

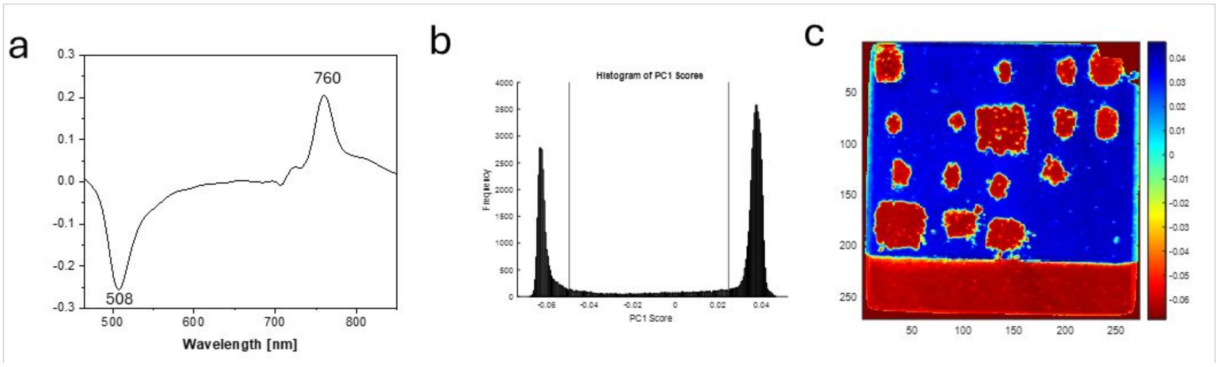

Figure S8. Results of PCA performed on the sample AlkB on AlkY. a) Loadings profile along PC1 b) Distribution of the score values along PC1, with identification of the thresholds (black lines) selected for the segmentation of the RGB score map. c) False color PC1 score map.

A stock solution of PY74 standard was prepared in dimethyl sulfoxide and a calibration curve was obtained (Figure S9) by plotting the integrated area values obtained for the peak at 24.6 min in the HPLC-DAD chromatograms extracted at the maximum of absorbance of 300 – 550 nm versus the PY74 concentration values in the 0.1 – 10 ppm range. The limit of detection (LOD) and limit of quantification (LOQ) of the method were calculated as the concentration corresponding to the blank average area plus 3 and 10 times its standard deviation, respectively. The instrumental LOD is 0.06 ppm, while the instrumental LOQ is 0.12 ppm.

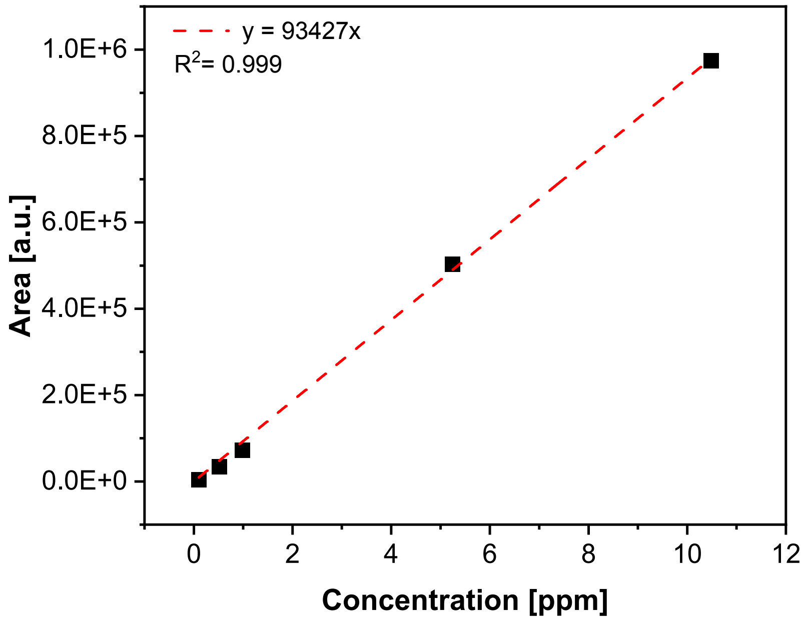

Figure S9. Calibration curve of PY74 in the 0.1-10 ppm range. The integrated area values were obtained from the HPLC-DAD chromatograms extracted at the maximum of absorbance of 300-550 nm.

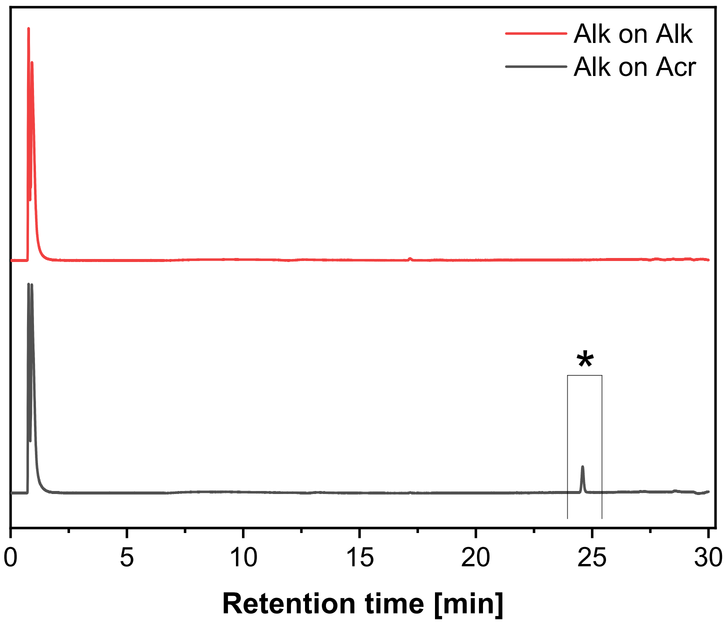

Figure S10. Representative HPLC-DAD chromatograms per each sample extracted at the maximum of absorbance in the 300-550 nm range (AlkB on AlkY in red, and AlkB on AcrY in black). * yellow pigment (PY74).

3. Reusability of photothermal electrospun mats

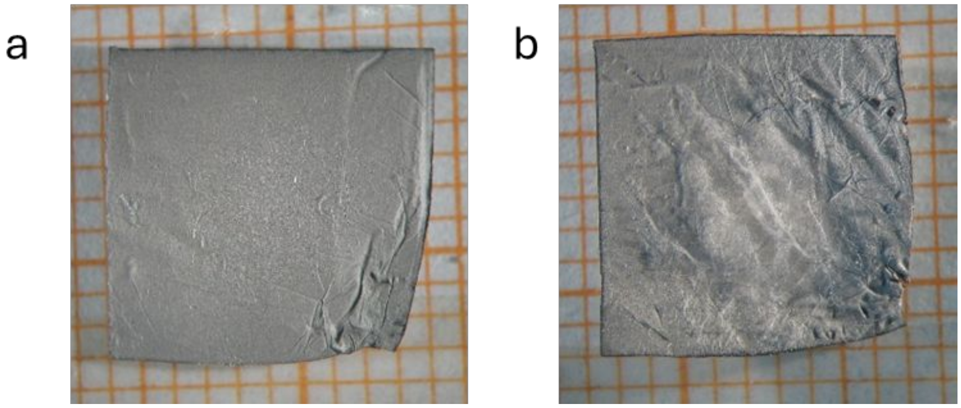

Figure S11. Photograph of the electrospun fabric before use (a) and after three reuse cycles (b).

Stress-strain tests were conducted using Linkam MFS350 microtensile stage equipped with a 20 N load cell, controlled by the accompanying Link software (Linkam Scientific Instruments, UK). The tests were carried out in traction mode with a load of 20 N at a crosshead speed of 10.00 mm min^{-1}. The tests specimens were of rectangular shape with a width of 5 mm, a gauge length of 1.5 cm, and a thickness in the range 20-50 μm; measurements were performed on at least 5 specimens from both the pristine nonwoven and the nonwoven after three cycles of use.

To reproduce the usage cycle on the sample geometry required for the mechanical tests, a nonwoven with dimensions of approximately 5 × 2.5 cm^2 was first saturated with solvent at the predetermined ratio of 2 μL mg^{-1}. Then, the wet nonwoven was applied on the mock-up and irradiated with red light. As the sample area is considerably larger than the red spot, a total of twelve irradiations, each lasting 2 min, were necessary to treat the entire sample area. Afterwards, the sample was dried, and the procedure was repeated two more times. The specimens for mechanical testing were subsequently sectioned from the treated sample.

A significant reduction in sample thickness (ca. 25%) was observed after three usage cycles. This compaction of the layers can be attributed to a decrease in interfiber spacing, leading to a reduction in overall porosity, while morphological changes in the fibers were excluded, as discussed in the main

text. Indeed, SEM analyses demonstrated that neither the fiber morphology nor the average fiber diameter changed after use. In addition, no significant variation in the macroscopic in-plane dimensions of the mats was observed. A control experiment was performed in which the nonwoven was immersed in GVL and dried without any irradiation treatment nor usage. Under these conditions, a comparable thickness reduction (~25%) was still observed, demonstrating that the compaction phenomenon is associated with solvent exposure itself rather than with the photothermal treatment process and its use.

Mechanical properties are typically evaluated by expressing stress as the applied force normalized to the cross-sectional area, while displacement is converted into strain by normalizing to the initial length. However, electrospun nonwovens are highly porous fibrous assemblies, and their apparent cross-sectional area includes a large fraction of void space. Consequently, the calculated stress strongly depends not only on the intrinsic properties of the fibers, but also on the porosity and packing density of the network. As previously mentioned, after solvent exposure, the mat undergoes a densification process associated with a reduction in thickness and interfiber spacing. Therefore, normalization of the force by the apparent cross-sectional area inevitably leads to higher apparent stress values after reuse. However, in this specific case, such an increase does not necessarily indicate that the fibers themselves became mechanically stronger, stiffer, or more brittle; rather, it primarily reflects the reduced void fraction and the denser packing of the fibrous network. In other words, the reduction in porosity occurring after three usage cycles renders a direct comparison of mechanical properties in terms of stress unreliable.

Consequently, the authors chose to report the mechanical response in terms of force rather than stress. Figure S12 shows representative force-strain curves, while Table S1 summarizes the average mechanical properties of the pristine nonwoven sample and the sample after three usage cycles. Despite the increase in the strain at break after three cycles of use, the force/strain ratio did not significantly change, demonstrating that the mechanical properties of the nonwoven are not compromised after up to three reuses. In other words, the force–strain analysis demonstrates that, under the same applied force, the pristine and reused mats undergo comparable deformation,

indicating that the practical mechanical robustness of the material is preserved after three reuse cycles despite the partial densification of the structure.

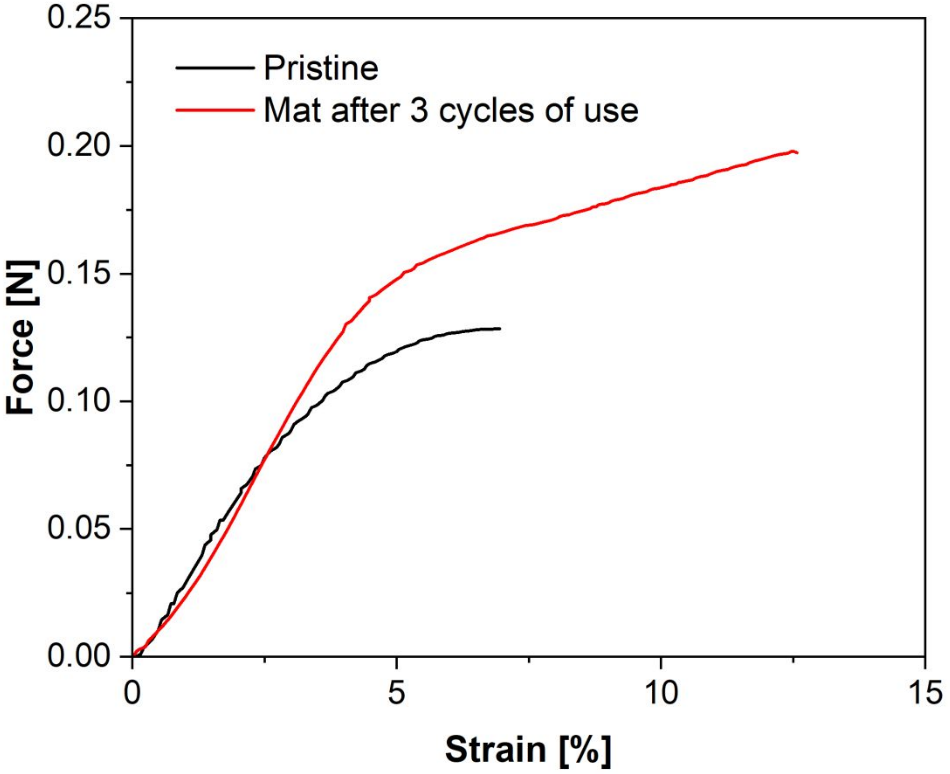

Figure S12. Representative force-strain curves of the pristine nonwoven and after three cycles of use.

Table S1. Mechanical data of the pristine nonwoven and after three cycles of use. Each result is reported with its standard deviation

**Sample**	**F/strain [N]**	**Force at break [N]**	**Strain at break [%]**	**Thickness [µm]**
Pristine	3.0 ± 0.1	0.13 ± 0.02	9 ± 3	42 ± 7
After three cycles of use	2.8 ± 0.7	0.191 ± 0.002	15 ± 7	32 ± 4

References

[1] Liu, Y., Ai, K., Liu, J., Deng, M., He, Y., & Lu, L., Dopamine-Melanin Colloidal Nanospheres: An Efficient Near-Infrared Photothermal Therapeutic Agent for In Vivo Cancer Therapy. *Adv. Mater.*, 25,1353-1359, 2013. https://doi.org/10.1002/adma.201204683

[2] Menichetti, A., Mordini, D., Rampazzo, E., Pane, A., Vicenzi, S., Petropoulos, V., Cerullo, G., Mancin, F., & Montalti, M., A simple method to distinguish light scattering from light absorption by nanoparticles, *Nanoscale Adv.*,7, 6786-6790, 2025. https://doi.org/10.1039/d5na00194c

[3] Nikitin, E. D., Popov, A. P., Bogatishcheva, N. S., & Faizullin, M. Z., Critical temperatures and pressures, heat capacities, and thermal diffusivities of ɣ-valerolactone and some alkyl pentanoates, *J. Chem. Thermodynamics* 149,106162, 2020.https://doi.org/10.1016/j.jct.2020.106162
